# Change in Nutritional and Biochemical Status in People Living with HIV-1 on Antiretroviral Therapy

**DOI:** 10.3390/v14112573

**Published:** 2022-11-20

**Authors:** Ranilda Gama de Souza, Sandra Souza Lima, Andresa Corrêa Pinto, Jacqueline Silva Souza, Tuane Carolina Ferreira Moura, Ednelza da Silva Graça Amoras, Luiz Fernando Almeida Machado, João Farias Guerreiro, Antonio Carlos Rosário Vallinoto, Maria Alice Freitas Queiroz, Ricardo Ishak

**Affiliations:** 1 Laboratory of Virology, Institute of Biological Sciences, Federal University of Pará (UFPA), Belém 66075-110, Brazil; 2 Graduate Program in Biology of Infectious and Parasitic Agents, Institute of Biological Sciences, Federal University of Pará (UFPA), Belém 66075-110, Brazil; 3 Human and Medical Genetics Laboratory, Institute of Biological Sciences, Federal University of Pará, Belém 66075-110, Brazil

**Keywords:** HIV-1, ART, nutritional profile, anthropometry, biochemical parameters, immunological markers

## Abstract

Antiretroviral therapy (ART) improves the quality of life of people living with HIV-1 (PLHIV) and reduces the mortality rate, but some individuals may develop metabolic abnormalities. This study evaluated changes in the nutritional status and biochemistry of PLHIV on antiretroviral therapy in a cohort that had not previously received ART and to follow up these individuals for 24 months after starting treatment. The initial cohort consisted of 110 individuals and ended with 42 people, assessed by a physical examination. A biochemical assay was performed using the colorimetric enzyme reaction technique, the proviral load was detected by qPCR and the quantification of the CD4/CD8 T lymphocytes was conducted by flow cytometry. PLHIV had increased levels of total cholesterol, LDL, triglycerides, ALT, urea and creatinine after 24 months of ART use (*p* < 0.05). In the assessment of the nutritional status, PLHIV had increased measures of Triciptal Skinfold, body mass index and arm circumference after the use of ART (*p* < 0.05). The viral load levels decreased and the CD4 levels increased after 24 months of ART use (*p* < 0.05). The change in the nutritional status in PLHIV on antiretroviral therapy seems to be a slow process, occurring in the long term, therefore, there is the need for a constant evaluation of these people to identify patients who need a nutritional intervention.

## 1. Introduction

An HIV-1 infection is an important cause of disease worldwide, and since the inception of AIDS, the impact on the human population has been of a great magnitude. By the end of 2020, there were approximately 37.7 million people infected with HIV-1 [1]. Brazil reported 381,793 new cases from 2007 to 2020, of which 36,218 were from the northern region of the country [2]. Brazil has one of the most successful public programs for the diagnosis of an infection, access to antiretroviral therapy (ART) and for the evaluation of the efficiency of ART by monitoring complementary tests and genotyping HIV-1 strains [3]. The establishment of ART prevents the progression of the disease, prolongs survival, improves ones quality of life and reduces the onset of opportunistic infections [4,5,6,7]. By June 2021, 28.2 million people had access to ART, which corresponds to 73% of all people living with HIV-1 (PLHIV) [1].

Despite the free access to ART in the Brazilian public health service, the success of the treatment can be influenced by several variables, starting with the appropriate choice of drugs, the frequency of the administration of antiretrovirals, the interference or dependence with the simultaneous ingestion of food, the side effects, the drug interactions and the psychosocial aspects of the patient, which are crucial for a patient’s adherence to therapy [4,8,9].

An HIV-1 infection without an ART intervention promotes weight loss due to the increased metabolic demands, which can lead to cachexia [10]. The nutritional status of an individual is influenced by the consumption and use of nutrients, and an inadequate intake may favor the development of diseases and hasten the progression to AIDS [11,12]. 

An HIV-1 infection and the use of ART are related to the development of metabolic and gastrointestinal changes, which can lead to malnutrition and a worsening of the clinical status of the patient [13,14,15]. Thus, an adequate nutritional treatment for PLHIV can help improve therapeutic and immune system responses [8,16,17]. The introduction of ART recovers the individual from the immunological weakness caused by the infection, but an insufficient caloric intake can have an impact on the efficiency of the treatment [18].

On the other hand, although treatment against an HIV infection improves the immune function and reduces the viral load, it may contribute to the development of diseases not related to AIDs, such as cardiovascular and kidney disease [19,20,21]. With the emergence of ART, there was a significant improvement in the quality of life, mainly due to a reduction in the mortality of PLHIV. However, there were also metabolic abnormalities (in sugars, lipids and dyslipidemia with direct consequences on the nutritional status of PLHIV) [15,22,23]. Nucleoside analog reverse transcriptase (NRTI) inhibitors have a high affinity for HIV-1 reverse transcriptase and inhibit human DNA polymerases, generating metabolic acidosis [24]. Protease inhibitors (IP) are more commonly associated with the appearance of dyslipidemia [25,26].

In this sense, it is important to assess the nutritional status of PLHIV before starting ART to identify individuals who need nutritional support, which acts as a complementary treatment to ART. In addition, the periodic monitoring of patients during treatment may allow for the identification of nutritional changes that may contribute to the development of metabolic diseases associated with the use of ART. Thus, the present study evaluated the change in the nutritional status, levels of biochemical markers, proviral load and quantification of CD4/CD8 T lymphocytes in people living with HIV before and after antiretroviral therapy.

## 2. Materials and Methods

### 2.1. Group Examined

An initial cohort was formed of 110 people of both sexes, aged 18 to 59 years, who tested positive for an HIV-1 infection and who were not yet on ART, attending the Specialized Care Service (SAE) Casa Dia in Belém/PA. The group was monitored from June 2016 to June 2018. The initial evaluation (T0; *n* = 104) occurred before the administration of ART; the cohort was evaluated a second time after 11–12 months of ART (T1; *n* = 69), and a third evaluation was performed after 24 months of ART (T2; *n* = 42). Among the 104 individuals selected, 42 were fully evaluated, attending the nutritional assessments and tests as scheduled. The rest were lost before the follow-up due to dropouts, deaths, absences at the evaluations and a lack of interest in adhering to ART.

In the evaluations, the epidemiological (demographic, social and economic) variables, laboratory markers (total cholesterol and fractions and triglycerides, ALT, AST, urea and creatinine), immunological factors (CD4+ and CD8+ T lymphocyte count), virologic markers (viral load quantification) and clinical nutrition markers (physical examination and anthropometry) were measured. The invitation to T1 and T2 was made by active search (phone, e-mail, SMS and WhatsApp, among others). The comparison of epidemiological and nutritional variables was performed with a group of 113 people from the university community, matched by sex and age, who were not infected with HIV-1.

### 2.2. Sample Collection

The blood samples (10 mL) were collected in Vacutainer tubes containing anticoagulant (K3-EDTA), packed in a thermal box for storage and transported to the Virus Laboratory of the Institute of Biological Sciences of the Federal University of Pará, where they were processed and separated into plasma and cell mass. The biological samples were stored at −20 °C until their use.

### 2.3. Physical Nutritional Examination and Anthropometry

The nutritional physical examination served as a basis for the nutritional diagnosis and to support dietary interventions. It involved a systemic and progressive evaluation, from head to toe, with the objective of determining nutritional deficiencies in regions such as the skin, mucous membranes, hair, eyes, lips, teeth, gums, tongue, trunk, upper and lower limbs [27,28,29]. Anthropometry is an indicator of the nutritional status and measures the body’s size and its proportions [30]. In this study, the following measures were taken:

(i) The current body weight was measured on a scale with a capacity of 150 kg (accuracy of 100 g; the two groups were weighed barefoot wearing light clothing, without accessories or adornments; the current weight (CW) was compared with the ideal weight (IW) and its usual weight (UW) for the classification of the individual in relation to a nutritional change).
(ii) The height was measured in meters by the technique described by Waitzberg (2001): using a metal stadiometer attached to the scale, without shoes or a hat, standing on the scale platform, with the heels together behind, and the body erect and the heels, buttocks, shoulders and head superficially touching the vertical wall of the measuring device with the line of sight facing the horizon [29].
(iii) Body mass index (BMI): a nutritional status indicator that followed the proposed calculation [31], accepted by the World Health Organization [32].
(iv) Triciptal skinfold (TS): the measurement of the skinfold thickness of the triceps, an appropriate method for determining body fat, was performed according to the procedure described by Augusto (1995) and Kamimura et al., (2005) [30,33]. Its interpretation was according to the standard of normality of Frisancho (1981), and the results were classified according to the reference values by Blackburn and Thornton (1979) [34,35].

### 2.4. Arm Circumference (AC)

The arm circumference represents the sum of the areas constituted by the bone, muscle and fat tissues of the arm and is an estimate of the total skeletal muscle protein [29,30]. Its interpretation was performed according to the standard of normality of Frisancho (1981), and the results were classified using the reference values of Blackburn and Thornton (1979) [34,35].

### 2.5. Arm Muscle Circumference (AMC)

The arm muscle circumference was used to determine muscle mass [30]. Its interpretation was performed according to the standard of normality of Frisancho (1981), and the results were classified using the reference values defined by Blackburn and Thornton (1979) [34,35].

### 2.6. Bioelectrical Bioimpedance Body Fat (%BF-BIA)

The bioelectrical impedance analysis (BIA) is an evaluation system of the body’s composition that consists of the passage of a low-intensity (500 to 800 µÄ) and high-frequency (50 kHz) electric current through the body, which is imperceptible by the patient. The MALTRON 906 bioimpedance device (Maltron, Rayleigh, Estex, UK) was used, measured and tested according to the manufacturer’s instructions. The reference values used to classify the percentage of body fat were obtained by the equipment from the variables included in it.

### 2.7. HIV-1 Plasma Viral Load

A viral load quantification was performed using real-time polymerase chain reaction (PCR) amplification technology, the Sample Purific CV HIV-1 extraction kit (Abbott) and the HIV-1 viral load amplification kit (Abbott, Chicago, IL, USA). The viral load results were expressed as copies/mL and log10.

### 2.8. Quantification of TCD4+ and CD8+ Lymphocytes

The CD4+ and CD8+ T lymphocyte counts were performed by flow cytometry (BD FACScaliburTM, Becton and Dickinson, San Jose, CA, USA) using the FACScountTM Reagents monitoring kit, following the protocol recommended by the manufacturer (Becton and Dickinson, San Jose, CA, USA). The results of the lymphocytes are expressed in cells/µL.

### 2.9. Biochemical Tests and Other Laboratory Tests

The measurements of the total cholesterol (TC), its fractions, low-density lipoprotein (LDL) and high-density lipoprotein (HDL) and triglycerides (TG), alanine aminotransferase (ALT), aspartate aminotransferase (AST), urea and creatinine were performed by the colorimetric enzymatic reaction technique, using the automated equipment Architect Abbott c8000 (Abbott Laboratories, IL, USA). 

### 2.10. Statistical Analysis

To describe the sociodemographic profile, descriptive statistics were used, the categorical variables were presented as frequencies and percentages and the numerical variables were expressed as the median and quartile deviation or the mean and standard deviation. The Kolmogorov–Smirnov test was performed to evaluate the normality and the Levene test was performed to evaluate the homogeneities of variances. To compare the laboratory and nutritional results obtained at T0, T1 and T2, an ANOVA was used for repeated measures and Tukey’s or Friedman’s post-test and Dunn’s post-test. The statistical analyses were performed using Prism software version 8, and a significance level of 5% was adopted.

## 3. Results

Table 1 shows the main epidemiological, laboratory and nutritional characteristics of the 42 participants evaluated in the study before starting ART (T0).

The analysis of the biochemical and immunological markers investigated showed that there were significant differences in the levels of all markers, with the exception of CD8^+^ T lymphocytes, between the periods evaluated (Table 2).

**Figure 1:**
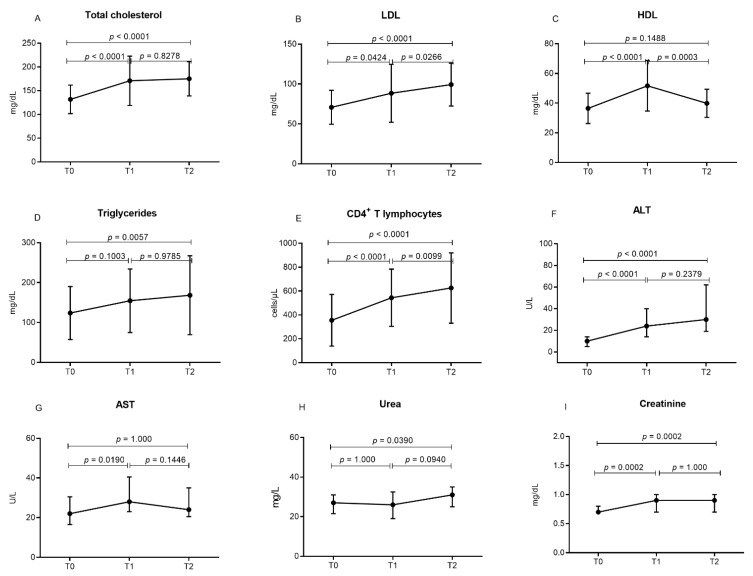
Comparison of the biochemical levels (**A**) total cholesterol, (**B**) LDL, (**C**) HDL, (**D**) triglycerides, (**E**) CD4^+^ T lymphocytes, (**F**) ALT, (**G**) AST, (**H**) urea and (**I**) creatinine of individuals with HIV1 between the periods evaluated. T0: initial assessment, before starting ART; T1: evaluation after 12 months of ART; T2: evaluation after 24 months of ART. Reference values: total cholesterol: <200 mg/dL; HDL:> 45 mg/dL; LDL: <110 mg/dL; triglycerides: <150 mg/dL; AST: 5 a 37 U/L; ALT: 7 a 41 U/L; urea: 20 a 40 mg/dL; creatinine 0,6 a 1,3 mg/dL.

Figure 1 demonstrates that the levels of total cholesterol, LDL, triglycerides, ALT and creatinine progressively increased (Figure 1A–C,F,I). The HDL cholesterol and AST levels increased after 12 months of ART use, but in the evaluation after 24 months of the treatment, there was a reduction (Figure 1C,G). The increase in urea levels was only observed after 24 months of ART (Figure 1H).

In the period prior to ART, all individuals had a viral load greater than 3 log10. After 12 months of ART (T1), this percentage decreased to 11.9%. In the second evaluation after the treatment (T2), only 7.1% had a viral load above log10 3, while the majority (88.1%) had no detectable viral load (Table 3).

**Figure 2:**
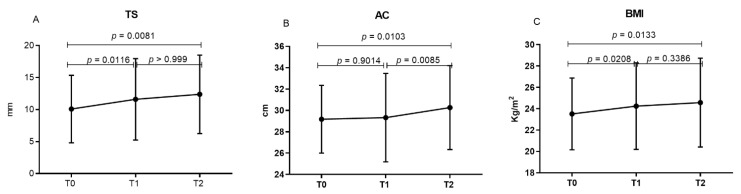
Comparison of the nutritional parameters (**A**) tricipital skinfold – TS, (**B**) arm circumference – AC and (**C**) body mass index – BMI of individuals with HIV1 between the periods evaluated. T0: initial assessment, before starting ART; T1: evaluation after 12 months of ART; T2: evaluation after 24 months of ART.

Of the nutritional parameters which were evaluated, the TS, BMI and AC showed significant differences in the mean/median values between the periods evaluated (Table 4). The TS and BMI measures progressively increased between the periods evaluated, without ART (T0), 12 months (T1) and 24 months (T2) after the use of ART (Figure 2A,C). The mean AC showed the greatest increase after the 24-month period of ART (Figure 2B).

Most PLHIV (88.1%) started and remained on the antiretroviral treatment regimen consisting of two nucleoside analogue reverse transcriptase inhibitors (NRTIs) and 1 non-nucleoside analogue reverse transcriptase inhibitor (NNRTI). One person (2.4%) started and remained with the regimen of two NRTIs and one protease inhibitor (PI) and four PLHIV changed their regimen over the evaluated periods (Table 5).

## 4. Discussion

Nutrition plays an important role in the current care of PLHIV and may have an impact on ART treatment; a poor nutritional status can impair the restoration of immunity [11,18]. Nutritional status and the progression of the human immunodeficiency virus (HIV) are intertwined [36]; an inadequate nutrition is associated with the pro-inflammatory environment of the infection, influencing the increase in overweight and obesity [37]. In addition, the persistence of HIV in tissue reservoirs may act in synergy with certain antiretroviral drugs and promote the increase in metabolic disorders. Lipodystrophy (altered fat breakdown) has been linked to NRTI and PI inhibitors, resulting in an increased total cholesterol, LDL and triglycerides, increasing the risk for cardiovascular disease and kidney disease [21,38].

The present study showed that PLHIV had increased levels of total cholesterol, LDL, triglycerides, ALT, urea and creatinine after 24 months of ART use. The reduced levels of HDL and the increase in the cholesterol and triglyceride levels is worrying, because although the levels have remained within the normal range, the continuous alteration in these markers can lead to the development of dyslipidemia. Obesity-related primary dyslipidemia is characterized by increased triglycerides, decreased HDL levels and an abnormal LDL composition [39]. Although a weight gain after the initiation of ART is associated with a reduced risk of mortality in low-weight and normal-weight individuals, the risk of metabolic diseases, including liver disease and cardiovascular disease, increases with excess adiposity [40].

The increase in the ALT, urea and creatine during the use of ART needs to be monitored in order to identify individuals who are at greater risk of developing liver or kidney disease. PLHIV with chronically elevated aminotransferase levels while on ART are at high risk of developing liver disease [41]. Several antiretroviral drugs have been associated with the progression of renal disease, the inhibition of renal tubular transporters that mediate a creatinine secretion or impaired phosphate reabsorption and low molecular weight proteins such as tenofovir and atazanavir can cause an acute tubular injury or tubular interstitial nephritis [42].

The evaluation of the immunological variables showed an increasing trend in CD4+ T lymphocytes, with a statistically significant difference when evaluated after 12 and 24 months of ART, and the reverse occurred in most cases regarding the viral load throughout the observation period. The highest percentage of participants had undetectable VL, indicating a good therapeutic approach, which confirms the importance of the adherence to treatment. The improvement in the immunological profile through the reduction in the viral load and increased levels of TCD4+ lymphocytes in PLHIV using ART is frequently observed [43,44,45,46,47]. The reduction in the viral replication and restoration of immunity are the main goals of ART, but the possible undesired effects related to the use of therapy cannot be neglected, so that PLHIV can have a controlled infection, without the emergence of possible metabolic changes.

In general, the evaluation of the means/medians of the nutritional parameters of the evaluated individuals showed a significant improvement in the nutritional profile throughout the observation period. TS are usually used as a simple and noninvasive method to evaluate the body fat reserves and, consequently, the amount of calories stored in the body [30]. In the present study, it was observed that the subcutaneous adipose tissue was inadequate in the three assessments for most PLHIV, characterizing lipodystrophy by atrophy, similar to what has already been described in the literature [48,49,50]. As the calorie stock changes slowly in malnutrition, this measure reflects a chronic inadequate food intake [51]. However, throughout the observation period, there was an increase in the ST measurements, portraying an increase in the body fat reserves in the group, which may be related to the consumption of hypercaloric foods or characteristics of the therapeutic regimen (most PLHIV used treatment containing NRTI, which induces an increase in cholesterol and triglycerides).

The protein reserves were observed from the measurements of AC [30], and the participants were classified as eutrophic in the three evaluations, similar to those already obtained [50,52]. However, there has also been evidence of malnutrition [49]. The AC represents the sum of the areas constituted by the fat, muscle and bone tissues; therefore, low AC values are related to a general nutritional deficit [51], which was not observed in this cohort.

BMI is an indicator for the diagnosis of nutritional status in epidemiological studies, considered an international standard through being adopted by the WHO, evaluating in a simple way whether the individual’s weight is adequate for their height and consequently their health condition, as it identifies possible malnutrition, being overweight or obesity [53,54]. Based on the BMI result, a more detailed anthropometric assessment can be started, considering other measures (the skinfold, arm, waist, etc.), which allows for a better identification of the type of alteration found [55]. In this way, the increase in the BMI, together with the increase in the CP and AC, showed a weight gain by PLHIV in the period of 24 months of ART use.

Although the nutritional parameters are in agreement with the reference measures of normality, the increase in the ST, cholesterol and triglycerides levels and BMI may be an indication that these individuals using ART need a follow-up to evaluate the possible metabolic changes.

Before the use of ART, PLHIV were commonly diagnosed with malnutrition and nutritional deficiencies [56], but this is no longer the case. The results show that most of the anthropometric parameters which were evaluated resulted in a normal weight, except for TS, which has shown the numbers of overweight PLHIV far surpass the cases of malnutrition [50,57,58]. Currently, weight gain, fat redistribution and obesity are new nutritional problems that PLHIV using HAART are presenting. Excess weight alone favors a series of social and psychological disorders, complicating the health status of individuals, which can lead to cardiovascular changes, hypertension and dyslipidemia, which can be aggravated by an HIV-1 infection. The use of ART associated with excess weight and a fat accumulation predisposes individuals to the development of metabolic syndrome [58]. Lipodystrophy is a common diagnosis in 13% to 62% of PLHIV [59]. Our results showed a subcutaneous fat deficit for most individuals after the analysis of the TS variable.

These data are important, since with the time of use of ART, the levels of CD4 T lymphocytes increase and the viral load decreases, but as this happens, the levels of lipids increase, showing that, in general, ART is effective and safe, but that individuals with a predisposition to metabolic diseases associated with nutritional changes need to be monitored so that they do not develop serious diseases, such as heart disease. 

The limitations of the study consisted of a loss of participants over the period which was evaluated, problems in the equipment used for the glucose measurement, which made it impossible to determine the blood glucose of several participants, and the study time since a follow-up over a longer time of PLHIV would allow a better understanding of the laboratory and nutritional parameters in relation to the use of ART.

## 5. Conclusions

In conclusion, although the use of ART promotes a rapid recovery in the immunological status of the patient, it seems to promote an increase in the levels of biochemical markers (cholesterol, triglycerides, urea and creatinine) and influence, suggesting, in a discrete way, a nutritional improvement of PLHIV. The change in nutritional status in PLHIV on ART appears to occur in the long term, so the constant evaluation of these individuals by specialized professionals is necessary to identify patients who need a nutritional intervention and/or are related to metabolic dysfunctions.

## Figures and Tables

**Table 1:** Epidemiological, laboratory and nutritional characterization of PLHIV before starting ART (T0).

Epidemiological Variables	*n*	%
Sex		
Male	30	71.4
Female	12	28.6
Age range		
19 to 29 years	21	50.0
30 to 39 years	15	35.7
40 to 57 years	6	14.3
Education		
Incomplete elementary school	6	14.3
Complete elementary school	3	7.1
Incomplete high school	3	7.1
Complete high school	21	50
Incomplete higher education	5	11.9
Complete higher education	4	9.6
Family income		
<1 minimum wage	4	9.6
1 to 3 minimum wages	33	78.5
4 to 6 minimum wages	4	9.6
No information	1	2.3
Marital status		
Single	26	62
Married	15	35.7
No information	1	2.3
Professionally active		
Yes	28	66.7
No	14	33.3
Smoking		
Yes	14	33.3
No	28	66.7
Alcoholism		
Yes	30	71.4
No	10	23.8
No information	2	4.8
Physical activity		
Yes	6	14.3
No	36	85.7
Comorbidities		
Yes	14	33.3
No	28	66.7
Laboratory variables	Median	IQR
Total Cholesterol	131.8 *	30.8 **
LDL	70.8 *	21.3 **
HDL	36.0	9.5
Triglycerides	109.5	78.8
AST	22.0	14.0
ALT	10.0	26.0
Urea	27.0	9.5
Creatinine	0.7	0.1
LTCD4^+^/µL	355.8 *	216.3 **
LTCD8^+^/µL	926.5	670.7
Nutritional status	Median	IQR
TS	10.8	9.7
AMC	25.7	4.6
BMI	23.5 *	3.4 **
AC	29.2 *	3.2 **
% BF-BIA	21.1 *	8.6 **

* Mean; ** Standard deviation; IQR: Interquartile range.

**Table 2:** Comparison of biochemical and immunological markers of PLHIV between the periods evaluated.

Variables	T0		T1		T2		
	Median	IQR	Median	IQR	Median	IQR	*p*-Value
**Biochemical**							
Total Cholesterol	131.8 *	30.8 **	171.0 *	51.9 **	175.1 *	36.2 **	<0.0001 ^a^
LDL	70.8 *	21.3 **	88.4 *	36.3 **	99. 3 *	26.8 **	<0.0001 ^a^
HDL	36.0	9.5	48.0	20.5	38.5	13.8	<0.0001 ^b^
Triglycerides	109.5	78.8	142.0	107.0	146.0	134.6	0.0062 ^b^
AST	22.0	14.0	28.0	7.5	24.0	14.5	0.0172 ^b^
ALT	10.0	26.0	24.0	26.0	30.0	43.0	<0.0001 ^b^
Urea	27.0	9.5	27.0	9.5	26.0	13.5	0.0234 ^b^
Creatinine	0.7	0.1	0.9	0.3	0.9	0.3	<0.0001 ^b^
**Immunological**							
LTCD4^+^/µL	355.8 *	216.3 **	544.1 *	239.3 **	626.1 *	294.5**	<0.0001 ^a^
LTCD8^+^/µL	926.5	670.7	811.0	417.0	796.0	529.7	0.7079 ^b^

* Mean; ** Standard deviation; IQR: Interquartile range; T0: initial assessment, before starting ART. T1: evaluation after 12 months of ART. T2: evaluation after 24 months of ART. ^a^ ANOVA test for repeated measures. ^b^ Friedman test.

**Table 3:** Viral load levels of PLHIV between the periods evaluated.

CVlog10—Copies/mL	T0 *n* (%)	T1 *n* (%)	T2 *n* (%)
<1.61	0 (0.0)	32 (76.2)	37 (88.1)
1.61–3.0	0 (0.0)	5 (11.9)	2 (4.8)
3.1–5.0	33 (78.6)	4 (9.5)	3 (7.1)
>5.0	9 (21.4)	1 (2.4)	0 (0.0)

T0: initial assessment, before starting ART. T1: evaluation after 12 months of ART. T2: evaluation after 24 months of ART. n: individuals’ number.

**Table 4:** Comparison of the nutritional parameters of PLHIV between the periods evaluated.

	T0		T1		T2		
	Median	IQR	Median	IQR	Median	IQR	*p*-Value
TS	10.8	9.7	11.6	8.6	12.1	8.4	0.0027 ^b^
AMC	25.7	4.6	25.8	5.6	26.6	5.3	0.0917 ^b^
BMI	23.5 *	3.4 **	24.2 *	4.1 **	24.6 *	4.2 **	0.0036 ^a^
AC	29.2 *	3.2 **	29.3 *	4.1 **	30.3 *	3.9 **	0.0028 ^a^
% BF-BIA	21.1 *	8.6 **	22.4 *	8.8 **	22.6 *	9.7 **	0.7164 ^a^

* Mean; ** Standard deviation; IQR: Interquartile range; T0: initial assessment; before starting ART. T1: evaluation after 12 months of ART. T2: evaluation after 24 months of ART. TS: triciptal skinfold; AMC: arm muscle circumference; BMI: body mass index; AC: arm circumference; % BF-BIA: bioelectrical bioimpedance Body Fat. ^a^ ANOVA test for repeated measures. ^b^ Friedman test.

**Table 5:** Distribution of PLHIV according to the antiretroviral therapy regimen used.

Therapeutic	Start of ART	T1	T2
Schemes	*n* = 42 (%)	*n* = 42 (%)	*n* = 42 (%)
ITRN + ITRNN	41 (97.6)	38 (90.5) *	37 (88.1) **
3TC + TDF + EFV			
ITRN + IP	01 (2.4)	04 (9.5)	03 (7.1) ***
3TC + TDF + ATV/r			
TRN + II + IP	-	-	01 (2.4)
3TC + TDF + DRV/r + DTG			
II + IP	-	-	01 (2.4)
DRV/r + DTG			

T1: evaluation after 12 months of ART. T2: evaluation after 24 months of ART. * three PLHIV changed treatment, one after 5 months and two after 11 months of ART (new regimen: TDF + EFV + ATV/r). ** a PLHIV changed treatment after 15 months of ART (new treatment: 3TC + TDF + DRV/r + DTG). *** a PLHIV changed treatment for the second time (initiated and performed treatment with 3TC + TDF + EFV for 11 months, then did 5 months of 3TC + TDF + ATV/r and changed to DRV/r + DTG. 3TC: lamivudina; TDF: tenofovir; EFV: efavirenz; ATV/r: atazanavir + ritonavir; DRV/r: Darunavir + ritonavir; DTG: Dolutegravir.

## Data Availability

The data analyzed in this study are included within the paper.
